# Mycorrhizal hyphae as ecological niche for highly specialized hypersymbionts – or just soil free-riders?

**DOI:** 10.3389/fpls.2013.00134

**Published:** 2013-05-16

**Authors:** Jan Jansa, Petra Bukovská, Milan Gryndler

**Affiliations:** Department of Ecology, Institute of Microbiology, Academy of Sciences of the Czech RepublicPraha, Czech Republic

**Keywords:** mycorrhizal symbiosis, hyphae-associated microbes, soil resources, mineral nutrients, carbon, hypersymbionts, theory

## Abstract

Mycorrhizal fungi interconnect two different kinds of environments, namely the plant roots with the surrounding soil. This widespread coexistence of plants and fungi has important consequences for plant mineral nutrition, water acquisition, carbon allocation, tolerance to abiotic and biotic stresses and interplant competition. Yet some current research indicates a number of important roles to be played by hyphae-associated microbes, in addition to the hyphae themselves, in foraging for and acquisition of soil resources and in transformation of organic carbon in the soil-plant systems. We critically review the available scientific evidence for the theory that the surface of mycorrhizal hyphae in soil is colonized by highly specialized microbial communities, and that these fulfill important functions in the ecology of mycorrhizal fungal hyphae such as accessing recalcitrant forms of mineral nutrients, and production of signaling and other compounds in the vicinity of the hyphae. The validity of another hypothesis will then be addressed, namely that the specific associative microbes are rewarded with exclusive access to fungal carbon, which would qualify them as hypersymbionts (i.e., symbionts of symbiotic mycorrhizal fungi). Thereafter, we ask whether recruitment of functionally different microbial assemblages by the hyphae is required under different soil conditions (questioning what evidence is available for such an effect), and we identify knowledge gaps requiring further attention.

## INTRODUCTION – MYCORRHIZAL SYMBIOSIS AND ITS HYPHAE-ASSOCIATIVE MICROBES

The association of plant roots with fungi has a very long evolutionary history (Remy et al., 1994; Berbee and Taylor, 2007) and can have different ecological outcomes, ranging from mutualistic, i.e., beneficial to both partners, to parasitic, i.e., beneficial to one partner and detrimental to the other partner (Johnson et al., 1997; Neuhauser and Fargione, 2004; Johnson and Graham, 2013). One of the oldest documented associations of “higher”^1^ plants with fungi is the arbuscular mycorrhizal (AM) symbiosis (Simon et al., 1993; Redecker et al., 2000). This type of association is established between more than a half of extant vascular plant species and members of a monophyletic and ancient group of soil fungi, the Glomeromycota (Schüßler et al., 2001). It is assumed that this symbiosis was established as a response to harsh environmental conditions at the time when the primitive plants were making their way from aquatic to terrestrial environments, providing them with major benefits in terms of facilitating nutrient acquisition from the primordial soils (Simon et al., 1993; Cairney, 2000; Taylor and Krings, 2005). During the evolution, some plant groups acquired fungi from sister clades (Ascomycota, Basidiomycota) as their mycorrhizal symbionts, establishing other kinds of mycorrhizal symbiosis such as ericoid, orchid, or ecto-mycorrhiza (Cairney, 2000). Some plants do establish more than one type of mycorrhizal symbiosis (e.g., arbuscular and ectomycorrhizal), whereas some few plant groups completely lost the capacity to establish any kind of mycorrhizal symbiosis (Wang and Qiu, 2006; Kariman et al., 2012).

**FIGURE 1:**
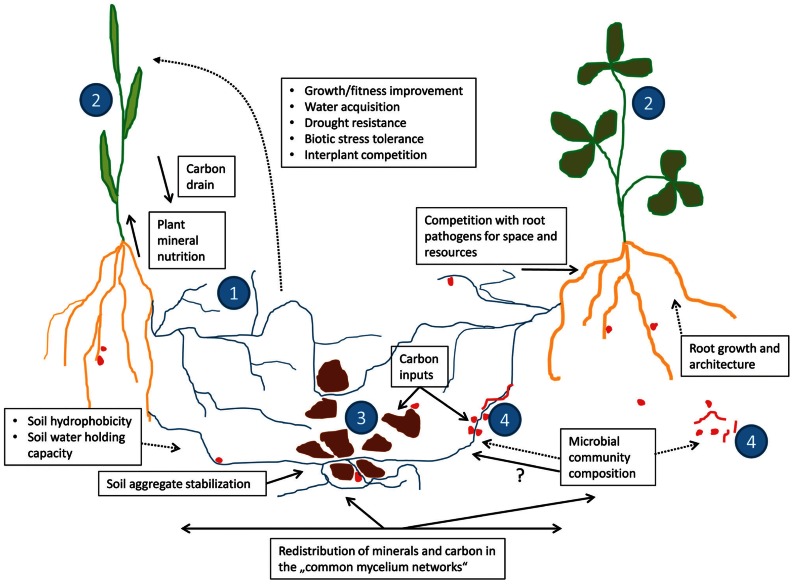
**Schematic representation of the different functions played by the arbuscular mycorrhizal (AM) fungi (1) in the physiology and ecology of their host plants (2)**. Mycorrhizal hyphae interconnect roots with soil particles (3), provide direct connections of root systems of different plant individuals (2), and interact with a number of soil microbes (4). Solid lines represent direct and the dotted lines indirect effects of the AM fungi on the plants, soil, and soil microbes.

The common feature of all types of mycorrhizal symbiosis is the fact that the fungi colonize two kinds of environment, namely the roots of the host plants (or, exceptionally, rhizoids or thalli of some bryophytes) and the surrounding soil, interconnecting these two habitats with their hyphae (Read et al., 2000; Jansa and Gryndler, 2010). This specific mode of fungal life is distinguishing the mycorrhizal fungi from root endophytes, which, although sometimes capable of spreading through or temporarily colonizing the soil, do not colonize both environments simultaneously for most of their life cycle (Faeth and Fagan, 2002; Hyde and Soytong, 2008; Jansa et al., 2011). Direct interconnection of soil with the roots through mycorrhizal fungi (**Figure 1**) is the basis for some of the most important functional features of the mycorrhizal symbiosis, namely the improved uptake of mineral nutrients and/or water from the soil by the host plants (Jakobsen, 1983; Jakobsen et al., 1992; Schweiger and Jakobsen, 2000; Drew et al., 2003; Augé, 2004; Allen, 2007; Martin et al., 2008). Such improvements have been frequently documented for a large number of host plants, soil and climatic conditions, mainly with respect to phosphorus, nitrogen as well as some micronutrients such as zinc and copper (Mosse, 1957; Smith and Read, 2008; Jansa et al., 2011).

Whereas the fungal hyphae inside the roots are mainly surrounded by plant cells, presenting quite a stable and homogeneous biotic environment, the hyphae extending to the soil are exposed to a great number of various biotic interactions (Jansa and Gryndler, 2010). The hyphae are challenged by diverse communities of soil prokaryotes, fungi, protozoans, nematodes, and other organisms. The composition of communities of soil microorganisms on the surface of mycorrhizal hyphae is usually quite different from the uncolonized (bulk, non-hyphospheric) soil, depending on fungal identity and possibly quite variable throughout the hyphae lifetime (Toljander et al., 2006; Scheublin et al., 2010; Izumi et al., 2013). For example, bacteria belonging to Oxalobacteraceae were established as a group with a specific aptitude to colonize the surface of AM hyphae (Scheublin et al., 2010), whereas *Burkholderia* and *Bradyrhizobium* were present on the ectomycorrhizal hyphae associated with pine trees (Timonen and Hurek, 2006; Kataoka et al., 2008). Various pure cultures of bacteria (e.g., *Rhizobium*, *Bacillus*, *Pseudomonas*) showed differential levels of attachment to the AM hyphae, depending on the AM fungal species and also the vitality of the hyphae (Toljander et al., 2006). Experimental evidence also exists for hyphal exudates of AM fungi having a pronounced effect on soil bacterial community composition, with some members of Enterobacteriaceae being particularly strongly promoted (Toljander et al., 2007). Very little direct evidence exists for association of mycorrhizal hyphae with eukaryotic organisms such as yeasts, although positive interaction between AM fungi and some yeasts with respect to the levels of root colonization were reported (Botha, 2011, and references therein).

The reasons behind recruiting of a specific microflora on the mycorrhizal hyphae remain mostly unclear – whether there are specific attractants or other signals involved, or whether the development of specific hyphosphere^2^ microbial communities is due to the release of other compounds by the hyphae (e.g., polysaccharides), remains speculative. We know, though, that the strength of association between AM hyphae and other microbes can be quite variable (Toljander et al., 2006; Jansa and Gryndler, 2010), ranging from loose/casual association to very tight, even intracellular mode of living (Ghignone et al., 2012). For most of the associations, the specific roles of the associative microbes in the fungal life and ecosystem processes still need to be established.

In this review we mainly focus on the AM fungi, because this is the most widespread type of mycorrhizal association. AM symbiosis has probably been the most challenging to study among all the mycorrhizal types due to the fact that the fungal partner cannot complete the life cycle without the host plant and (for most of the fungal taxa) also without the soil environment. The knowledge on this specific biological system has therefore been slower to accumulate than in other mycorrhizal types. Yet this knowledge is particularly relevant for many natural ecosystems as well as for most agricultural production systems, vegetated by plants reliant on the AM symbiosis for their nutrition and stress tolerance. Here we collate the available scientific knowledge on the identity and putative roles of AM fungal hyphae-associated microbes in relation to the mycorrhizal fungi and also to the mycorrhiza-host plants. More specifically, we analyze the potential involvement of the microbes in nutrient cycling and carbon (C) transformation in the AM fungal hyphosphere.

## FUNCTIONS OF THE ASSOCIATIVE MICROBES

Improved acquisition through the mycorrhizal host plants (as compared to the non-mycorrhizal plants) of orthophosphate and other mineral nutrients with limited diffusion in soil (e.g., Zn^2^^+^) has been sufficiently explained by the hyphae gathering the nutrients beyond the root depletion zone (Li et al., 1991; Jakobsen et al., 1992; Jansa et al., 2003; Schnepf and Roose, 2006; Thonar et al., 2011). However, improvements of uptake of highly mobile nutrients such as N in the form of nitrate or ammonium (Mäder et al., 2000; Scherer and Frost, 2004; Tanaka and Yano, 2005; Miransari, 2011; Fellbaum et al., 2012) and acquisition of nutrients bound in organic forms (Jansa et al., 2011, and references therein) have been much more difficult to explain. For example, Hodge et al. (2001) and Hodge (2003) reported increased rates of mineralization of N bound in plant residues in the presence of an AM fungus, and Koide and Kabir (2000) reported acquisition of P by the AM hyphae from organic forms in an *in vitro* system. This compounded previous reports on AM fungal acquisition of phosphorus from organic sources in soil (Tarafdar and Marschner, 1994; Feng et al., 2003). These findings have, however, sometimes been difficult to replicate and/or interpret (Joner and Jakobsen, 1995; Hodge et al., 2000; Hodge, 2001). Furthermore, the metabolic capacity of AM fungi to release phosphorus from organic molecules has been questioned (Joner and Jakobsen, 1995; Joner et al., 2000). Thus there are different niches where hyphae-associative soil microbes (either prokaryotes, yeasts or filamentous fungi, alone, or together with their grazers such as collembolans, nematodes, or amoebas) could step in and play important roles in nutrient cycling and plant nutrition (Joner and Jakobsen, 1995; Leigh et al., 2011).

### MINERALIZATION OF ORGANIC NUTRIENTS

Mineralization of organic nutrients seems to be primarily conducted by associative microbes such as bacteria (e.g., actinomycetes) and/or fungi, rather than the AM fungi themselves. This is quite different from other mycorrhizal types, where the mycosymbionts recruit from fungal groups possessing effective degrading pathways for complex organic compounds (e.g., Basidiomycota) and where axenic cultures provided unequivocal proof of their degrading capacity (Bending and Read, 1997; Read et al., 2004). There is limited evidence that the AM-hyphae associative prokaryotes are responsible for the degradation of organic materials in the vicinity of the AM hyphae to extract the nutrients or energy or both, and the AM hyphae can then take the mineral nutrients released to the soil solution (Leigh et al., 2011; Herman et al., 2012). The AM fungi are thus priming the degradation of organic nutrients in soil through inducing activity of specific microbes in their hyphosphere (Talbot et al., 2008). In this respect, eukaryotic associative microbes (e.g., basidiomycetous yeasts such as *Cryptococcus* or *Rhodotorula*) are particularly interesting as these were previously shown (1) to be closely associated with AM spores and hyphae, (2) they enhance the development of mycorrhizal structures in host plant roots, and (3) they also possess specific enzymatic activities enabling degradation of complex organic molecules (Alonso et al., 2008; Boby et al., 2008; Botha, 2011). Depending on the requirements of the hyphae-associative microbes (they may need either the nutrients or the carbon, or both) these nutrients can be regarded as the desired product or a waste. In any case, AM hyphae can take up these nutrients when released to the soil solution, either directly competing with the degraders or using the surplus of the nutrients released by the associative microbes during their search for energy.

### PRODUCTION OF BIOACTIVE COMPOUNDS

Some of the microbes on hyphal surface can also be involved in production of signaling, antibiotic and/or allelopathic compounds. There are relatively few details known on producers of such bioactive compounds on the surface of AM hyphae, especially because most of the microbes have not yet been cultured and their community composition is just becoming uncovered (Scheublin et al., 2010). In spite of this lack of information, there is circumstantial evidence that many of the microbes present in the AM fungal hyphosphere are producing bioactive compounds (Hoffman and Arnold, 2010; Bidondo et al., 2011; Seipke et al., 2012). For example, the presence of living microbes usually had much stronger effect on the growth of AM hyphae out of root sections under axenic conditions than many of the tested pure compounds with known signaling function, such as plant growth regulators (Gryndler et al., 1998) or flavonoids (Gryndler and Hršelová, 1998). Furthermore, there are microorganisms identified as “mycorrhiza helper bacteria” that, upon co-inoculation with the AM fungi, increase the colonization rates of the host roots (Garbaye, 1994; Frey-Klett et al., 2007; Bonfante and Anca, 2009). Production of bioactive compounds by hyphae-associated microbes could also explain some of the effects of plant–plant interactions as the hyphal networks have been shown to transfer the allelopathics over large distances in soil (Barto et al., 2011).

### PRODUCTION OF RECALCITRANT ORGANIC (GLOMALIN-LIKE) COMPOUNDS

Some years ago, the AM fungi were assumed to produce an elusive recalcitrant glycoprotein called glomalin, which was predicted to serve as a glue sticking soil particles in aggregates, holding soil water back, and potentially increasing bioavailability of mineral nutrients, among other functions (Wright et al., 2000; Millner and Wright, 2002; Rillig, 2004; Treseder and Turner, 2007). It seems, however, that glomalin is in fact a whole group of organic compounds of unclear biological origin, some of which may well originate from the AM fungi, but then it is chewed and transformed by a number of other organisms in the soil (Gadkar and Rillig, 2006; Whiffen et al., 2007; Janos et al., 2008; Sousa et al., 2012). It is quite likely that microbes on hyphal surfaces contribute greatly to the transformations of these compounds (Bolliger et al., 2008; Gonzalez-Chavez et al., 2008), although the exact pathways and reaction rates are still unknown.

### TRANSFORMATION OF RECALCITRANT ORGANIC COMPOUNDS

Along similar lines, AM hyphae-associated microbes are also likely, one way or another, to participate in oxidative polymerization of humic compounds (Piccolo et al., 2000). This process in soil is facilitated by a number of microbes producing oxidizing enzymes (Chefetz et al., 1998; Sinsabaugh, 2010; Zavarzina, 2010), and is usually wrapped under the term “humification.” Not well defined due to a variety of organic compounds involved, humification is ecologically an extremely important process of long-term stabilization of soil organic matter. Although the AM-induced humification is unlikely to fully revert the catabolic processes leading to release of mineral nutrients and energy bound in the soil organic matter (Laheurte et al., 1990; Cheng et al., 2012), it is definitely a subject worth further attention, not only from carbon sequestration point of view, but also in the light of potential industrial applications (Jeon et al., 2012).

### ATMOSPHERIC DINITROGEN FIXATION

Atmospheric dinitrogen fixation is an ecologically important function fulfilled solely by prokaryotes. Although there is little information on increased incidence of diazotrophic bacteria on the surfaces of AM fungi, there are studies showing that hyphae of some other (e.g., ectomycorrhizal) fungi do host such bacteria and that this may be important for nitrogen nutrition of the mycorrhizal plants such as pines (Paul et al., 2007) and/or for ripening of truffle fruitbodies (Gryndler et al., 2013, and references therein).

## CARBON ALLOCATION TO THE ASSOCIATIVE MICROBES

Nearly all organic carbon^3^ in the soil originates from the photosynthesis carried out either by plants or by photosynthetic prokaryotes, one way or the other. The carbon fixed by the plants is first distributed throughout the plant body and a significant portion, between 4 and 30% of the net photosynthesis production, is transferred to the AM symbionts (Paul and Kucey, 1981; Jakobsen and Rosendahl, 1990; Drigo et al., 2010; Lendenmann et al., 2011; Calderon et al., 2012). This movement from the plant to the fungus is usually quite fast, taking just a few hours (Johnson et al., 2002; Staddon et al., 2003; Olsson and Johnson, 2005; Leake et al., 2006). Thereafter, within hours to days the carbon is either built into the hyphal structures, respired, or making its way through other members of the hypho- or rhizosphere (Jones et al., 2004; Leake et al., 2006; Kramer et al., 2012). Drigo et al. (2010) demonstrated fast movement of C from the plants to the AM hyphae and thereafter a gradual transfer of the carbon to *Burkholderia* and *Pseudomonas*, likely the hyphae-associative microbes. In contrast, no appreciable allocation of C was observed to *Bacillus *and Actinobacteria. In another experiment it was shown that, upon the presence of AM fungal hyphae in ^13^C-labeled organic patches, fatty acid biomarkers for a number of prokaryotic groups were less enriched in ^13^C than those in patches not colonized by the AM fungi (Herman et al., 2012). This indicates that (at least some) of the prokaryotes derived their C preferentially from the AM fungi rather than from the plant litter. How is the C directed toward the hyphae-associated microbes is not completely known, but it has been hypothesized that trehalose released by the AM hyphae or other hyphal exudates may play a role (Bago et al., 1999; Drigo et al., 2010).

**FIGURE 2:**
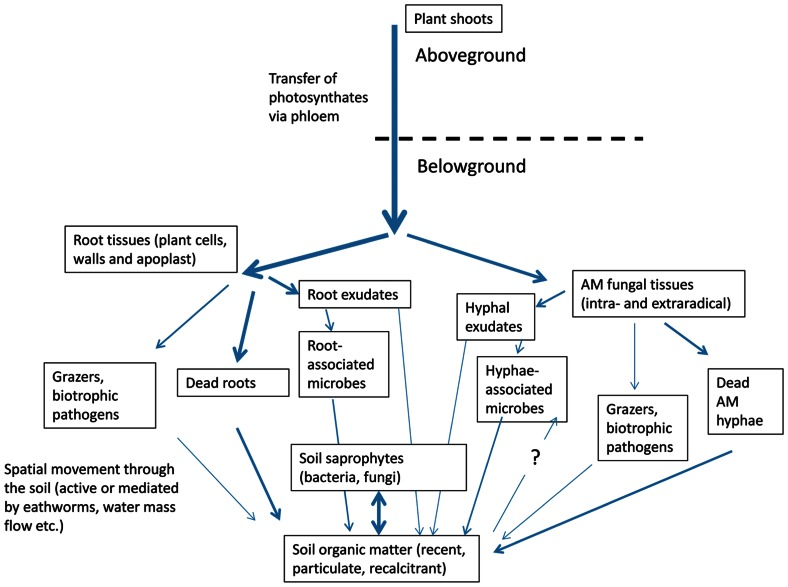
**Pathways of photosynthetically fixed carbon redistribution in the belowground compartment of the plant-fungal-soil system**. Thickness of lines represents approximate volume/rate of fluxes. Respiration losses associated with every step and inputs through aboveground litter are not shown here.

An alternative pathway of the C moving from plants to the hyphae-associated microbes is through the decay of dead AM hyphae or through grazing on living hyphae (**Figure 2**). These processes can be rather fast, especially given that the half-life of some of the terminal hyphae is just a few days (Staddon et al., 2003). However, cell walls of the hyphae are unlikely to be degraded fast, and, because the active cytoplasm is usually retracted to the backbone hyphae upon death of the terminal hyphal branches (Bago et al., 1998; Logi et al., 1998), there is not much fast food left for the degraders. On the other hand, specialized grazers on the hyphae can get access to the living cytoplasm, redistributing the hyphal cell content/carbon throughout the soil on short time scales (Fitter and Garbaye, 1994; Klironomos and Ursic, 1998).

Still another pathway for the plant C to get into the soil is through the root cell products (exudates) or dead root cells or biomass transferred to grazing/parasitic animals or microbes (**Figure 2**). These can also move through the soil and this movement can effectively mix a large soil volume. This mixing can be so intensive that it can effectively disable observation of spatially discrete processes such as localized transfer of C from the hyphae to associated microbes.

Under the condition that some hyphae-associated microbes get direct access to fungal C, e.g., in forms of hyphal exudates (Artursson and Jansson, 2003; Toljander et al., 2007) and, at the same time, they fulfill functions beneficial for the AM fungus or the associated plant, such co-existence could be classified as hypersymbiosis (Starr, 1975). However, to the best of our knowledge, unequivocal proof of hypersymbiosis still needs to be established in this case, especially because the identity of the different microbes could not yet be directly linked to their functions *in situ*.

## DYNAMICS OF THE ASSOCIATIONS UNDER FLUCTUATING ENVIRONMENTAL CONDITIONS

Changing ecosystem-wide environmental conditions (e.g., temperature, humidity, atmospheric CO_2_ levels) will likely change a great number of ecosystem parameters including the size and composition of soil microbial communities, routes of C fluxes, rates and pathways of organic nutrient recycling, and ecosystem productivity (St Clair and Lynch, 2010; Cheng et al., 2012; Gutknecht et al., 2012; Zavalloni et al., 2012; Drigo et al., 2013). In soil, environmental conditions can also change dramatically on a small spatial scale, for example through deposition of organic materials such as plant litter or dung, local disturbance through burrowing animal activities and the like (Freymann et al., 2010; Stromberger et al., 2012).

Response of AM fungi to fluctuation of soil conditions and also how the benefits of the host plants derived from the mycorrhizal symbiosis vary upon changing the environmental conditions are the subject of research in a number of ongoing studies (Drigo et al., 2010; Hawkes et al., 2011; Gavito and Azcon-Aguilar, 2012; Gutknecht et al., 2012; Drigo et al., 2013). However, how stable is the association of AM fungi with their hyphae-associated microbes when exposed to different or changing environmental conditions, whether the composition and/or function of the associative microbes shifts depending on the quality of organic materials in the hyphal vicinity, has not yet been explicitly addressed.

## FURTHER RESEARCH NEEDS

Obviously, association of AM hyphae with specific microbes is potentially explaining many unexpected, contradictory, and poorly replicable observations in the past. One of the most fascinating quests of mycorrhizal ecology is now to determine if these microbes are metabolically associated with AM fungal hyphae (i.e., deriving their C exclusively or mainly from the hyphae) or whether they derive their energy mainly from mineralization of soil organic matter. The first scenario would qualify these prokaryotes as hypersymbionts, which would add further level of complexity in our understanding of symbiotic world, whereas the second scenario would advocate for a theory of facultative associations. So far it is not possible to unequivocally declare any of the microbes found in the AM fungal hyphosphere as hypersymbionts, although preliminary evidence suggests preferential C flow from the hyphae to certain rhizosphere bacteria (Drigo et al., 2010). At the same time, however, strong evidence is missing for any direct benefits of these very microbes to their fungal hosts.

A second very interesting story is how resistant is this association to the fluctuations of environmental conditions. Do the AM fungi recruit different microbial community on their hyphae depending on the specific soil conditions, or is the identity of the microbes rather stable, and just their function adapts, e.g., when submitted to different soil conditions such as organic patches? Is it thus beneficial to develop mechanisms to vertically transmit the associative microbes to next generations or is the community established always anew, after the spore germinates and/or the secondary mycelium develops?

There is currently a whole range of methods allowing unprecedented precision and high throughput data production (e.g., next generation sequencing and proteomic analyses). Using stable and radioactive isotopes allows quantification of fluxes of carbon and mineral nutrients, and even the organisms involved in some of the processes (i.e., stable isotope probing for tracing the pathways of C fluxing). However, these methods, regardless of their novelty and precision, need to be applied in smartly designed experiments, with proper controls and with sufficient number of replicates/gradient coverage. Thus proper design of the experiments addressing the open questions is fully as important as the proper use of the available analytical tools.

The studies of hyphae-associated microorganisms will have to take into account the variability and dynamic behavior of the soil as the environment for the life of microbial community. An interdisciplinary approach involving the viewpoints of soil chemistry, physics, population biology, mycology, and plant physiology will probably be unavoidable to receive reliable understanding of the role played by the inhabitants of AM hyphae surfaces.

## Conflict of Interest Statement

The authors declare that the research was conducted in the absence of any commercial or financial relationships that could be construed as a potential conflict of interest.

## Footnotes

1 Multicellular plants, often developing vascular tissues and specialized organs such as leaves, stems, and roots. Unlike others, we also count bryophytes and liverworts to this group, in spite of the fact that they sometimes develop neither vascular tissues nor specialized organs.

2 Soil volume under direct influence of AM fungal hyphae.

3 A small fraction of the carbon in the soil organic matter can originate from non-photosynthetic fixation of CO_2_ in specific metabolic pathways (e.g., Krebs cycle) in both autotrophs and heterotrophs. In heterotrophs, it is the energy fixed up during photosynthesis, which feeds these biochemical reactions, but the carbon moiety can originate either from the sugars built up in photosynthesis or from the CO_2_ directly fixed by the heterotrophs.
